# Management of Dysphagia Pre- and Postoperatively in a Case of Eagle's Syndrome

**DOI:** 10.1155/2015/305736

**Published:** 2015-03-17

**Authors:** Vicki Lewis, Bari Hoffman Ruddy, Jeffrey Lehman, Erin Silverman, Brian Spector

**Affiliations:** ^1^The Ear, Nose, Throat and Plastic Surgery Associates, Orlando, FL 32806, USA; ^2^Department of Communication Science and Disorders, University of Central Florida, Orlando, FL 32816, USA; ^3^Department of Physiological Sciences, University of Florida, Gainesville, FL 32610, USA

## Abstract

Eagle's syndrome (ES) is rare condition, most frequently described within the context of case study presentation. ES results from elongation of the styloid process, contributing to symptoms such as globus sensation in the throat, as well as pain localized to the ear, neck, face, or tongue. Additional symptoms can include hypersalivation, change in vocal quality, submandibular swelling, and dysphagia. This report discusses evaluation, diagnosis, and surgical intervention with respect to Eagle's Syndrome in a patient presenting with moderate-severe dysphagia.

## 1. Introduction

Eagle's syndrome (ES) is a rare condition, first identified in 1937 by Dr. Watt W. Eagle, an otolaryngologist after whom the disorder is named [1]. ES results from abnormal length (greater than 3 cm) and positioning of the styloid process and is estimated to affect as many as 4–7% of the general population [1, 2]. Of these, only approximately 4–10% will experience symptoms related to the condition [1, 2]. ES sometimes presents following trauma or tonsillectomy [1, 3–6]. However, in many cases, the etiology of ES cannot be identified. Common presenting symptoms of ES may include throat, face, tongue, or ear pain or globus sensation, hypersalivation, voice changes, submandibular swelling, and dysphagia [2, 3]. While most published case reports review surgical outcomes associated with ES, this report details a combined surgical-behavioral approach to the rehabilitation of pharyngeal swallow function pre- to (up to six months) postoperatively.

## 2. Case Report

This 59-year-old male patient presented to our otolaryngology practice upon referral from his primary care physician for further evaluation of chronic dysphagia that began nine years prior, occurred primarily with solids, and was accompanied by intermittent neck pain and recurrent pneumonia. The patient underwent additional diagnostic procedures one year prior to his presentation to our practice, including computed tomography (CT) of the neck, esophagogastroduodenoscopy (EGD), and videofluoroscopic swallowing assessment (VFSS). Neck CT revealed anterior cervical osteophytes and asymmetry of the submandibular glands. EGD findings were normal. VFSS was abnormal with subsequent recommendation for a nonoral means of nutrition (NPO) due to aspiration risk. The patient was noncompliant with this recommendation and continued to consume a regular consistency diet with thin liquids. Upon presentation to our practice, fiberoptic endoscopic evaluation of swallowing (FEES) was completed in order to visualize the pharyngeal and laryngeal structures and to further evaluate the pharyngeal phase of the swallow. FEES revealed apparent narrowing of the hypopharynx and moderate-severe pharyngeal dysphagia evident with moderate residue in the valleculae and pyriform sinuses and along the posterior pharyngeal upon administration of thicker viscosities. Additionally, supraglottic penetration of thin liquid, puree, and mechanical soft consistencies was evident during and after the swallow. The patient exhibited inconsistent sensation for the penetrant.

**Figure 1:**
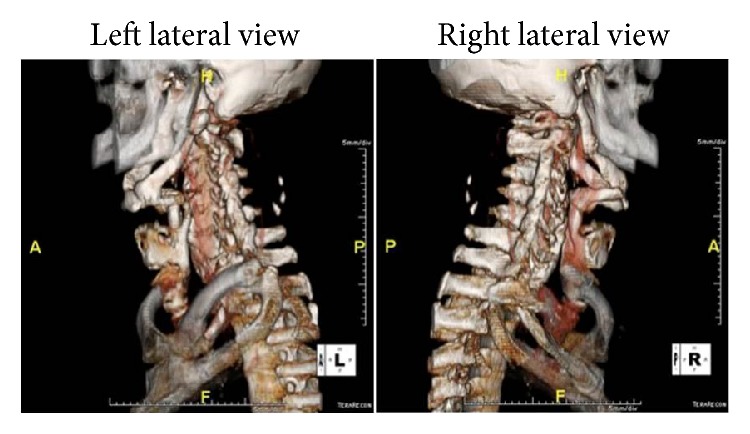
CT scan of the neck with 3-dimensional reconstruction of the larynx.

Given the abnormal findings on FEES, a CT scan of the neck with 3D reconstruction of the laryngeal framework was requested. CT revealed enlargement of styloid process bilaterally. The hyoid bone was enlarged with pseudoarticulation to the superior cornu of the thyroid cartilage bilaterally. This pseudoarticulation served to deform the right posterior pharyngeal mucosa at the level of the epiglottis, yielding a diagnostic impression of Eagle syndrome. Further examination revealed ankylosis between the thyroid cartilage and hyoid, as well as along the stylohyoid ligament and extending into the skull base/styloid process. The ligament appeared ossified and markedly thickened, suggestive of severe ES presentation (Figure 1).

## 3. Surgical Approach

Traditional surgical approaches for ES create a large neck incision. However, in this case, a shorter (4 cm) incision was made at the level of the hyoid for subsequent neck exploration with bilateral stylohyoid ligament and bone resection. The centrally located suprahyoid muscle bridge was left intact and neck dissection was completed lateral to the central segment of the hyoid. Approximately 15 mm of ossified stylohyoid ligament was removed bilaterally beginning at the lesser cornu of the hyoid bone in order to release the ankylosis. Palpation of the larynx following resection revealed significant increases in lateral and vertical laryngeal mobility. On postop day one, repeat VFSS was completed and revealed evidence of persistent, significant pharyngeal phase dysphagia. The patient exhibited silent penetration to the level of the true vocal folds (TVF) both before and after swallow on 3–5 cc puree trials administered via spoon. These dysphagia symptoms were observed to persist postoperatively and a percutaneous endoscopic gastrostomy tube (PEG) was placed on postop day three. Pharyngeal swallowing exercises were introduced by a speech-language pathologist shortly afterward, during the patient's inpatient stay. These tasks targeted increased laryngeal elevation and pharyngeal constriction [7].

During this time, the patient remained NPO with nutrition administered via PEG. A repeat FEES was completed on postop day 18. During both the preop and postop examinations, the Penetration Aspiration Scale [8] (PAS; Table 1) was used to describe swallow physiology during presentations of multiple boluses of varying consistencies.

At both the pre- and postoperative FEES exams, multiple trials of thin liquid (3 cc and 5 cc), nectar-thick liquid (5 cc), and puree consistencies (5 cc) were administered via spoon, revealing improved laryngeal elevation and pharyngeal constriction when comparing postoperative to preoperative FEES findings. This resulted in improved pharyngeal clearance of the bolus.  Table 2 presents the pre- and postoperative Penetration Aspiration Scale or PAS [8] scale scores for this patient. Note that with virtually all consistencies (thin liquid, nectar thick liquid, thin puree, thick puree, and mechanical soft) the patient demonstrated clear improvement over preoperative scores. Solids were not administered preoperatively and therefore cannot be compared with the postoperative findings, which were excellent. Additional improvements were observed in postswallow pharyngeal residue within the left vallecular and left pyriform sinus/cricoid region on thin and nectar thick liquids. Persistent, mild pharyngeal residue was evident after swallow of puree consistencies but cleared partially with cues to reswallow. Following repeat FEES, oral trials of thin and nectar thick liquid were initiated although the patient continued to receive primary nutritional support via PEG.

At this point, the pharyngeal strengthening exercises initiated previously were shaped into a home-based dysphagia rehabilitation protocol by the speech-language pathologist, once again targeting laryngeal elevation and pharyngeal constriction [7]. The patient returned for repeat FEES approximately once every 4 weeks on an outpatient basis in order to provide ongoing monitoring of swallowing function with diet consistency upgrade as appropriate. A puree consistency diet with thin liquids was initiated 34 days postop with resumption of a full regular consistency diet with thin liquids and discontinuation of PEG assisted feedings at 66 days postop. Continued, gradual improvement in airway protection and pharyngeal constriction was noted over repeat FEES examinations with marked improvements in pharyngeal residue after swallow evidenced secondary to improved laryngeal elevation and pharyngeal constriction.

Clinically, these improvements were attributed to the surgical intervention. Removal of a portion of the calcified stylohyoid ligament appeared to have decreased ankylosis, thereby allowing for improved laryngeal excursion during swallow. Improvements in laryngeal mobility also contributed to enhanced airway protection and postswallow pharyngeal clearance. Prior to surgery, laryngeal tethering precluded activation of muscles responsible for laryngeal elevation. An additional, unexpected benefit of improved pharyngeal and laryngeal sensation emerged postsurgically. Over the long term, the patient's pharyngeal function continued to improve, although never to the point where no dysphagia symptoms were evident. At 6 months postop the patient continued to tolerate a regular consistency diet with no clinical signs of airway compromise during intake. Anecdotally, the patient reported greater ease of swallowing and ability to comfortably consume a larger variety and volume of foods with improved quality of life. Because the surgical approach involved a smaller incision than is typically used for ES, minimal neck scarring was evident postoperatively.

## 4. Discussion 

Although dysphagia is a reported symptom of ES, particularly in the context of carotid artery involvement [3], little published information exists as to the extent and nature of these symptoms; therefore comprehensive and ongoing assessment of dysphagia symptoms is critically important. Following surgery to remove stylohyoid calcification and elongation, ankylosis was significantly improved upon laryngeal palpation. In spite of this, the patient's swallow function did not immediately improve, characteristic of delayed improvement in pharyngeal dysphagia following surgery which appears to be common in these patients. Introduction of pharyngeal swallowing exercises targeting increased laryngeal elevation and pharyngeal constriction during the swallow directly preceded observable improvement in swallow, including improvements in postswallow pharyngeal clearance and airway penetration. These improvements emerged beginning approximately one month postop with continued improvement noted over time. Ultimately, the patient was able to discontinue nonoral feedings, eventually resuming a full regular consistency diet with thin liquids approximately at two months after operation.

## 5. Conclusion

This case underscores the importance of a* combined modality* (surgical and behavioral) approach to dysphagia rehabilitation. It is likely that restricted range of motion of the pharyngeal and laryngeal musculature prior to surgical release contributed to disuse resulting in muscular weakness. Resolution of ankylosis did not immediately resolve the dysphagia symptoms; however it did allow for improved swallow function over time resulting from muscle strengthening and practice. Pre- and postoperative evaluation of swallow function is necessary in order to address issues surrounding swallow safety, airway protection, and nutrition.

## Figures and Tables

**Table 1:** The 8-point Penetration Aspiration Scale [8].

	Score	Description of events
$\overset{⃡}{\text{Abnormal}\;\;\text{Normal}}$	1	Material does not enter the airway.
	2	Material enters the airway, remains above the vocal folds, and is ejected from the airway.
	3	Material enters the airway, remains above the vocal folds, and is not ejected from the airway.
	4	Material enters the airway, contacts the vocal folds, and is ejected from the airway.
	5	Material enters the airway, contacts the vocal folds, and is not ejected from the airway.
	6	Material enters the airway, passes below the vocal folds, and is ejected into the larynx or upper airway.
	7	Material enters the airway, passes below the vocal folds, and is not ejected into the larynx or upper airway.
	8	Material enters the airway, passes below the vocal folds, and no effort is made to eject (e.g., “silent aspiration”).

**Table 2:** PAS [8] scores obtained during FEES examination preoperatively and postoperatively. Multiple scores for various consistencies represent multiple presentations of that same consistency.

Consistencies presented	Preoperative FEES	Final postoperative FEES
Thin liquid	4, 4	1, 1
Nectar thick liquid	1, 1	1, 1
Thin puree (applesauce)	7, 8	1, 1
Thick puree (pudding)	3, 3	1, 1, 2
Mechanical soft consistency	3, 2	1
Solid consistency	Not administered	1, 2
